# Depression in silent lacunar infarction: a cross-sectional study of its association with location of silent lacunar infarction and vascular risk factors

**DOI:** 10.1007/s10072-014-1794-5

**Published:** 2014-04-22

**Authors:** Ri-Han Wu, Qiang Li, Yan Tan, Xue-Yuan Liu, Jing Huang

**Affiliations:** Department of Neurology, Shanghai Tenth People’s Hospital of Tongji University, 301# of Middle Yanchang Rd, Zhabei District, Shanghai, China

**Keywords:** Silent lacunar infarction, Depression, Vascular risk factors, Body mass index, Inflammation, Physical activity

## Abstract

Most previous studies reported a close link between fresh infarcts and post-stroke depression. However, studies on the relation of depression and silent lacunar infarction (SLI) are limited. This study aims to analyze the effects of SLI and the vascular risk factors on depression. A total of 243 patients with SLI were divided into depression and non-depression groups. The presence and location of SLI were evaluated with magnetic resonance imaging. Depression was assessed with the Patient Health Questionnaire-9 and vascular risks factors were collected. We used *t* tests and *χ* ^2^ test to compare the baseline characteristics of the two groups and the multivariate logistic regression model to identify the risk factors for depression. Univariate analysis results showed that the proportion of patients with SLI in basal ganglia was significantly higher in the depression group (65.0 versus 32.8 %; *P* < 0.001) than in the non-depression group, and multiple prevalent factors had significant differences between the two groups. However, on multivariate logistic analysis, some of these factors were eliminated, and SLI in basal ganglia remained an independent predictor of depression with an odds ratio of 3.128 (*P* = 0.018). In addition, vascular risk factors, including high body mass index level, presence of inflammation markers (e.g., CRP, TNF-α, Hs-CRP, and IL-6), and lack of physical activity, were associated with depression. Our findings suggest that SLI in basal ganglia is associated with a higher risk of depression. Vascular risk factors, which are intertwined, may propose the pathological basis of depression in SLI.

## Introduction

Silent lacunar infarction (SLI) has commonly been regarded as a benign subtype of stroke, associated with relatively mild disability. Most previous studies reported a close link between fresh infarcts and post-stroke depression (PSD). PSD is defined as ‘depression occurring in the context of a clinically apparent stroke, which is opposed to silent cerebral vascular disease which belongs to vascular depression [1]. Vascular depression was originally hypothesized to be chronic and persisting. Compared to vascular depression, the course of PSD seems even more complex and dependent on timing of onset [2]. The role of vascular risk factors in the etiology of PSD and vascular depression is less obvious than it appears. As we known, hypertension, atrial fibrillation, smoking, diabetes, and cardiovascular disease are premorbid vascular risk factors with clear longitudinal association with cerebrovascular disease, however, previous study found strong positive association between vascular burden and frequency of depression among patients without stroke, but not among patients with stroke [3]. These different findings in the etiologies of PSD and vascular depression may suggest an independent etiologic process for each depressive syndrome.

Limited information is available on the detailed outcome of depression in SLI [4]. Recent study postulated that progressive accumulation of microvascular lesions may promote depression [5]. The presence of lacunar infarction (LI) in subcortical regions, namely in the thalamus, basal ganglia, and deep white matter has been shown to promote depression [6]. Another study only found an association of LI in deep white matter with depressive symptoms [7]. Differences in inclusion criteria, diagnostic protocols of LI, and assessment of depression may contribute to such different viewpoints in the above studies. However, it remains unclear whether SLI can cause depression through these locations. Recent study has also suggested that chronic vascular burden may contribute significantly to the pathogenesis of depression [8]. The prevalence of vascular risk factors has been empirically linked with the onset and severity of depression in both cross-sectional and longitudinal studies [9]. Conditions such as hypertension [10], diabetes [11], obesity [12, 13], inflammation [14], extent of physical activity [15], and serum lipid profile [16] had been associated with depression in the general population. However, only few previous studies have examined the influence of these risk factors on depression in a population of patients diagnosed with SLI. In the present study, we performed a cross-sectional study focusing on the location of SLI and the related risk factors to explore the relationship between SLI and depression.

## Materials and methods

### Participants

A cross-sectional study was performed on the patients attending to the Department of Neurology in 10th People’s Hospital at Shanghai from September 2012 to November 2013, mainly with non-specific symptoms such as headache, dizziness, vertigo and limb numbness, simultaneously without any localized neurological signs. The study has been approved by the institutional review boards and all participants have provided written consent for participation. A total of 272 participants diagnosed with SLI through magnetic resonance imaging (MRI) were consecutively recruited in our study and attributed to depression and non-depression groups, respectively. The number of focal lesions of SLI should be between three and five in each location. Patients with histories of stroke, vascular dementia, heart failure with a New York Heart Association degree 3 or 4, myocardial infarction, atrial fibrillation, rheumatic valvular heart disease, arteritis during this study were excluded. Patients with any Grade of leukoaraiosis according to Fazekas’ scale and fresh infarction identified through MRI were also excluded.

### Measurement

All images were analyzed by two experienced radiologists blinded to the clinical data. SLI was defined as focal lesions ranging from 3 to 15 mm in diameter, with hypointensity on T1-weighted images, hyperintensity on T2-weighted images. At the time, the location of the SLI was recorded as thalamus, basal ganglia, deep white matter, or brain stem.

Age, gender, education (less than high school/high school or college/graduate degree), smoking habits (smoking or non-smoking), alcohol intake (yes or no), history of diabetes mellitus, and coronary heart disease were recorded for each subject during enrolment. Systolic and diastolic blood pressures (mmHg) were recorded according to the average levels in the last 3 months by questionnaire. Each participant’s height and weight were measured without shoes and heavy clothing. The body mass index (BMI) was calculated as the ratio of weight in kilograms to the height in square meters (kg/m^2^). The participants were grouped into three categories according to their BMI value. Based on WHO recommended BMI cutoffs for Asian populations [17], the participants were considered as underweight to normal for BMI values <23 kg/m^2^, overweight for BMI values ≥23.0–27.5 kg/m^2^, and obese for BMI values ≥27.5 kg/m^2^. Waist and hip circumference was measured and waist–hip ratio (WHR) was calculated. Physical activity was determined by the question: “How much exercise you did in the last 6 months, i.e., have you exercised for 15–20 min of brisk walking, swimming, general conditioning, or recreational sports?” Possible answers included not at all active, a little active (1–2 times/month), fairly active (3–4 times/month), quite active (1–2 times/week), very active (3–4 times/week), or extremely active (≥5 times/week). The participants were grouped into three categories according to the activity level: inactive, light or moderately active, and vigorously active.

The fasting blood samples from the participants were obtained at around 7:00 am and were analyzed at the clinical laboratory department of the Shanghai 10th People’s Hospital of Tongji University. The blood samples were assessed for the following markers of inflammation: C-reactive protein (CRP), high-sensitivity C-reactive protein (hs-CRP), interleukin-6 (IL-6), and tumor necrosis factor alpha (TNF-α). Additionally, insulin, total cholesterol, triglycerides, high-density lipoprotein cholesterol (HDL-C), and low-density lipoprotein cholesterol (LDL-C) were also measured.

In the present study, diagnosis of depression in all patients was established during a face-to-face interview mainly held by Dr. Ri-Han Wu and Dr. Qiang Li, two of the physician in our department of neurology. Depression was assessed with the Patient Health Questionnaire (PHQ)-9 [18], a 9-item scale that assesses the presence of 9 DSM-IV symptoms for major depressive disorder. Responses were scored on a 4-point scale from 0 to 3, indicating that the participant experienced the symptom “not at all” “on several days” “on more than half the days” or “nearly every day” with higher scores indicating more severe symptoms. Scores on this scale were categorized as none to minimal (PHQ score 0–3), mild to moderate (PHQ score 4–9), or severe (PHQ score ≥10) [18]. Participants with the PHQ scores ≥4 were attributed to depression group and the PHQ scores 0–3 were attributed to non-depression group.

### Data analysis

All the data were analyzed with SPSS (version 18.0, SPSS, Inc., Chicago, Ill., USA). Baseline characteristics of the patients are shown in Table 1. Continuous variables were assessed as mean ± SD and categorical variables were assessed as counts in percentage. To compare the baseline characteristics, continuous variables were analyzed using *t* tests and categorical variables were analyzed using *χ* ^2^ test. Only the variables with *P* < 0.05 were included in the multivariate logistic regression model. Odds ratio (OR) and 95 % confidence intervals (CIs) were obtained in models that adjusted for potential confounders. Consistency tests about the radiological diagnosis between two radiologists were performed by calculating intra-class correlation coefficient (ICC). Consistency tests about the PHQ scale between two physicians were performed by Kappa test. *P* < 0.05 was considered to be statistically significant difference.

**Table 1:** Baseline descriptive statistics for all subjects and by depression status

	Non-depression (*n* = 183)	Depression (*n* = 60)	*P* value
Age (years)	67.16 (9.09)	66.45 (9.95)	0.203
Gender			**0.013**
Men	81 (44.3 %)	15 (25.0 %)	
Women	102 (55.7 %)	45 (75.0 %)	
Smoker	42 (23.0 %)	9 (15.0 %)	0.259
Alcohol consumption	21 (11.5 %)	6 (10.0 %)	0.752
Education			**0.044**
Less than high school/high school	152 (83.6 %)	58 (95.0 %)	
College and graduate degree	30 (16.4 %)	3 (5.0 %)	
Physical activity			**<0.001**
None	51 (27.9 %)	33 (55.0 %)	
Mild to moderate	60 (32.8 %)	9 (15.0 %)	
Vigorous	72 (39.3 %)	18 (30.0 %)	
BMI (kg/m^2^)			**<0.001**
<23	97 (53.0 %)	15 (25.0 %)	
23–27.5	63 (34.4 %)	27 (45.0 %)	
>27.5	24 (13.1 %)	18 (30.0 %)	
WHR [M(SD)]^a^	0.98 (0.08)	0.96 (0.08)	0.089
Systolic BP (mmHg) [M(SD)]	137.05 (15.43)	135.10 (15.90)	0.400
Diastolic BP (mmHg) [M(SD)]	77.43 (9.91)	75.25 (7.00)	0.135
Coronary artery disease	39 (21.3 %)	15 (25.0 %)	0.551
Diabetes	50 (27.9 %)	18 (30.0 %)	0.751
Fasting insulin (mIU/L) [M(SD)]	13.00 (20.93)	11.34 (10.04)	0.554
Blood lipid (mg/dl) [M(SD)]			
Total cholesterol	4.88 (1.13)	5.14 (1.09)	0.120
Triglycerides	1.43 (0.67)	1.49 (0.79)	0.549
HDL-C	1.25 (0.42)	1.35 (0.39)	0.105
LDL-C	2.97 (0.94)	3.12 (0.96)	0.287
Inflammation markers [M(SD)]			
CRP (mg/L)	7.14 (2.34)	8.80 (2.19)	**<0.001**
Hs-CRP (mg/L)	2.14 (2.76)	3.53 (3.41)	**0.005**
IL-6 (pg/ml)	3.28 (1.80)	4.22 (2.53)	**0.002**
TNF-α (pg/ml)	11.54 (4.56)	17.02 (5.37)	**<0.001**
Location of lacunar infarction			
Thalamus	50 (27.9 %)	19 (30.0 %)	0.879
Basal ganglia	60 (32.8 %)	39 (65.0 %)	**<0.001**
Deep white matter	68 (37.2 %)	18 (30.0 %)	0.395
Brain stem	53 (29.0 %)	15 (25.0 %)	0.612

The *P* values <0.05 are in bold

^a^ *M*(SD), mean (standard deviation)

## Results

A total of 243 participants met the entry criteria and formed the study sample. The Kappa value was 0.923, indicating significant consistency between two physicians about the diagnosis of depression. The ICC value for different locations of SLI namely thalamus, basal ganglia, deep white matter, brain stem was 0.972, 0.946, 0.962 and 0.953, respectively, indicating significant consistency between two radiologists about the radiological diagnosis. The baseline characteristics of the total sample are shown in Table 1. Sixty (24.7 %) of the 243 participants were classified as depressed. Women and those with high school or less than high school education showed more symptoms of depression. Both overweight and obese patients and physically inactive participants displayed more symptoms of depression. Those with high levels of inflammation markers including CRP, hs-CRP, IL-6, and TNF-α also showed depressive symptoms. The proportion of the patients with SLI in basal ganglia was significantly higher in the depression group compared with the non-depression group. No significant statistical differences were noticed within WHR, blood pressure, blood lipid levels, coronary artery disease, diabetes, fasting insulin, or other locations of SLI between the depression and non-depression groups.

**Fig. 1:**
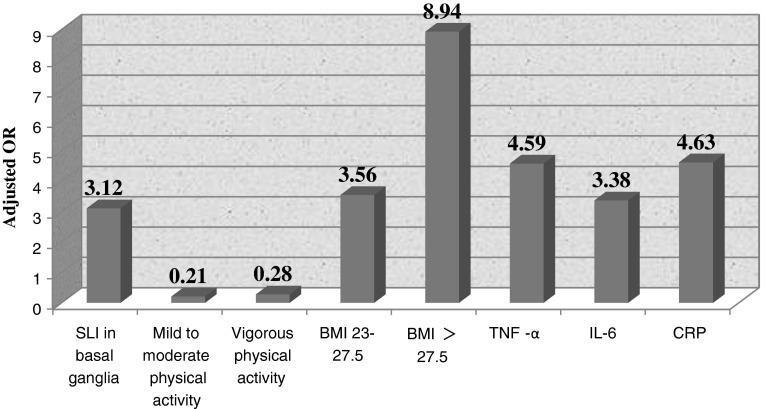
Adjusted OR value by each variables which were statistically significant, namely SLI in basal ganglia, mild to moderate/vigorous physical activity, BMI, TNF-α, IL-6 and CRP

The following variables were entered into the multivariate logistic model: gender, education, physical activity, BMI, inflammation markers, and SLI in basal ganglia. The results of multivariate logistic regression were shown in Fig. 1 according to the adjusted OR value of each variables which were statistically significant. SLI in basal ganglia was a significant independent imaging predictor of depression with an OR of 3.12 (95 % CI: 1.221–8.015). Compared with complete lack of physical activity, mild to moderate (OR: 0.21; 95 % CI: 0.05–0.82), and vigorous physically active (OR: 0.28; 95 % CI: 0.10–0.80) group showed strong association with depression. Compared with normal weight, overweight (OR: 3.56; 95 % CI: 1.27–9.94), and obese (OR: 8.94; 95 % CI: 2.41–33.17) population displayed more depression. Additionally, all inflammation markers were significantly associated with depression. Those with high levels of CRP (OR: 4.63; 95 % CI: 1.85–11.61), TNF-α (OR: 4.59; 95 % CI: 1.91–11.06), IL-6 (OR: 3.38; 95 % CI: 1.29–8.90), and hs-CRP (OR: 1.5; 95 % CI: 0.58–3.85) exhibited more depressive symptoms compared with those of normal levels. However, gender, and education were no longer associated with depression.

## Discussion

In our study of 243 patients, 24.7 % of the participants were classified as depressed. After considering a set of possible confounding factors, we found multiple prevalent factors that affected depression. In particular, SLI in basal ganglia and vascular risk factors including higher BMI levels, and inflammation markers, CRP, TNF-α, hs-CRP, and IL-6 can increase the risk for depression. Physical activity on the other hand can protect against depression.

Despite the fact that LI is found in 10–30 % of elderly individuals with 6 % of newly affected cases annually, they are thought to be clinically silent in the majority of elderly cases [19]. Majority of the previous neuroimaging studies have reported a close link between small subcortical fresh infarcts and PSD; however, only limited studies have examined the effect of SLI in depression-related disorders [6]. The main biological mechanism of PSD is the ischemic lesion interrupted the neural fiber projections ascending from the midbrain to the brainstem, which leaded to a decreased bioavailability of biogenic amines, namely serotonin (5HT), dopamine (DA), norepinephrine (NE) and acetylcholine [20]. In case of SLIs, the damage to small vessels supplying subcortical pathways disrupts the neurotransmitter circuitry that is involved in mood regulation in a chronic pattern. When the accumulation of the infarcts exceeds a certain threshold, patient could become more vulnerable to developing depression. Some putative mechanisms suggest that lesions at various sites may result in depression through direct disruption of the cortico-striato-pallido-thalamo-cortical (CSPTC) circuits or their modulating systems [21]. Our findings indicate that SLI in basal ganglia can increase the risk of depression, which is partly consistent with the results of the previous study [6]. Given that basal ganglia consist of many central gray matter nuclei, cortico-basal ganglia circuitry dysfunction may play a role in the neurobiology of depression. Cortico-basal ganglia circuitry consists of segregated functional subcircuits and their fibers project from specific functional regions of the cortex to specific nuclei in the basal ganglia [22]. These fibers include monoamine and glutamatergic fibers, which are involved in the mood disorder. Interruption of the monoamine neurotransmitter fibers results in the fluctuation of serotonin and norepinephrine levels in the orbitofrontal pathway and the prefrontal lobe [21]. In addition, damage in the glutamatergic routes disrupts glutamatergic transmission, which is also associated with depression [23].

Alexopoulos et al. proposed the theory of “vascular depression” to suggest that pathologies to the vessels play a unique role in the onset of depression [21]. He also proposed a threshold model. In this model, vascular risk factors may lead to depression only after crossing a certain threshold. Such relationships could be cumulative following linear or curvilinear patterns. The threshold model can account for multifactorial contributions to depression. In addition, this model allows for each risk factor alone or in combination to cross the threshold to induce neural circuit changes, thereby mediating depression. Thus, the processes of these pathways could all contribute to depression both independently and collaboratively [24]. By conducting univariate analysis, we found that multiple prevalent factors affected depression in our study. However, on multivariable analysis, we found that some of these effects were eliminated, and other effects remained associated with depression. This finding indicates that risk factors are intertwined, thereby promoting the pathological basis of depression in SLI.

Since, we found vascular risk factors included obesity, inflammation, and lack of physical activity were associated with depression, their underlying mechanism may be as follows. Obesity may induce hypothalamic–pituitary–adrenalaxis (HPA axis) dysregulation, which is associated with depression [25, 26]. Abnormalities to the inflammatory system of both depressed and obese people were observed [14], providing possible biological explanations for the association between depression and obesity. In our study, we found that both obesity and inflammation markers were risk factors for depression. Increase in physical activity is also linked to the reactivity of the HPA axis and is associated with lower levels of inflammatory markers [27]. Our finding suggests that engaging in both mild to moderate and vigorous physical activity acts as protective factor in depression, which is in line with existing research. Inflammation is associated with altered brain function in areas mediating depression [28]. Our data on the effect of inflammation markers on depression are consistent with these findings [29, 30]. Several mechanisms may explain these relationships. Pro-inflammatory cytokines reduce the availability of serotonin [31]. Furthermore, pro-inflammatory cytokines may lead to a reduced hippocampal volume [32], which is also seen in depression [32]. This result may indicate that treatment with the anti-inflammatory agent may result in a significant decrease in depressive symptoms [33].

The present study had several limitations. First, our data were gathered by self-reported questionnaires, which could have led to bias by patients over or underreporting physical activity, depression, past history, or educational attainment. The fact that these individuals have agreed to participate in a clinical trial may suggest different emotional and behavioral characteristics, which may impact the generalizability of our results concerning depression. However, our prevalence estimates were similar to those previously reported studies on LI [6]. Second, our cross-sectional study was restricted to the participants with a baseline depression score. We did not examine the longitudinal course of depression over time. Finally, we cannot draw firm conclusions regarding the direction of causality between the progression of structural changes and depressive symptoms, because depressive symptoms were only assessed at the follow-up study.

## Conclusion

In conclusion, our findings suggest that SLI in basal ganglia and vascular risk factors including BMI, inflammation markers, and physical activity is strongly associated with depression. Furthermore, these risk factors are clearly intertwined, proposing a pathological basis of depression following SLI. By demonstrating such mechanisms affect neural circuit function, this study opens a door for future research to further examine the mechanisms that can improve the outcome for depression [24]. It is critical that patients with SLI are screened early for depression and are provided with appropriate treatment.
